# Meeting ethical challenges with authenticity when engaging patients and families in end-of-life and palliative care research: a qualitative study

**DOI:** 10.1186/s12904-022-00964-x

**Published:** 2022-05-16

**Authors:** Matthew DeCamp, Ahmed Alasmar, Stacy Fischer, Jean S. Kutner

**Affiliations:** 1 Center for Bioethics and Humanities and Division of General Internal Medicine, Department of Medicine, University of Colorado Anschutz Medical Campus, 13080 E. 19th Ave, Mail Stop B137, Aurora, CO 80045 USA; 2 Center for Bioethics and Humanities, University of Colorado Anschutz Medical Campus, Aurora, USA; 3 Division of General Internal Medicine, Department of Medicine, University of Colorado School of Medicine, Anschutz Medical Campus, Aurora, USA; 4 Division of General Internal Medicine, Department of Medicine, University of Colorado School of Medicine, Anschutz Medical Campus, Aurora, USA

**Keywords:** Patient and family engagement, Palliative care research, End-of-life care, Ethics, Professionalism

## Abstract

**Background:**

Delivering high quality, patient- and family-centered care depends upon high quality end-of-life and palliative care (EOLPC) research. Engaging patients and families as advisors, partners, or co-investigators throughout the research lifecycle is widely regarded as critical to ensuring high quality research. Engagement is not only an ethical obligation, it also raises ethical challenges of its own. We conducted a qualitative study to understand ethical challenges and potential solutions when engaging patients and families in EOLPC research.

**Methods:**

We recruited and interviewed 20 clinical investigators and 22 patients or family caregivers through the Palliative Care Research Cooperative Group (PCRC). Interview transcripts were analyzed using constructivist grounded theory methodology. Analysis sought to identify ethical challenges and potential solutions, as well as to synthesize findings into practical recommendations tailored to engaging patients and families in EOLPC research.

**Results:**

Our study identified 8 ethical challenges considered unique to the EOLPC research context and 11 potential solutions to these challenges. The most frequently described ethical challenges included the need to minimize burdens of engagement for patients and caregivers, challenges of dealing with death and illness, and paternalism or “gatekeeping” (i.e., withholding the opportunity to participate from patients or caregivers). Investigators and patients or family caregivers conceptualized ethics challenges differently; several issues appeared to fall outside a traditional research ethics paradigm and more into the ethics of relationships. We synthesized these findings into 4 practical recommendations hypothesized to support authentic engagement.

**Conclusions:**

Engaging patients and families in EOLPC research can raise unique ethical challenges. These challenges can be overcome to empower participation, minimize the unique burdens of EOLPC, and promote diversity. Whereas traditional research ethics tend to emphasize protecting research participants who may be vulnerable, an ethics approach based on authentic engagement that explores what it means for investigators and patients or family caregivers to be in a relationship may be needed. Future research is needed to explore this approach and test these recommendations in practice.

**Supplementary Information:**

The online version contains supplementary material available at 10.1186/s12904-022-00964-x.

## Background

Palliative care distinctly strives to achieve the best quality of life for patients who are experiencing serious illness and their family members and caregivers. Estimates suggest that two-thirds of all people in the United States could benefit from palliative care at some point, and this estimate does not include the family members and caregivers who may also benefit [1, 2]. Delivering high-quality, evidence-based patient- and family-centered palliative care depends upon the conduct of rigorous end-of-life and palliative care (EOLPC) research [3].

Engagement with patients and families throughout the research lifecycle is critical to ensuring it addresses the needs, priorities, and values of patients and families. The importance of this engagement has been long recognized by the Institute of Medicine (now the National Academy of Medicine), US Patient-Centered Outcomes Research Institute (PCORI), the National Institutes of Health and others [3–6]. Evidence is accumulating that engagement improves research and health outcomes by ensuring research is responsive to patients’ preferences, beliefs, and values, as well as by enhancing research recruitment and dissemination of research [4, 5, 7].

Although engagement is widely regarded as an ethical best practice [5, 8], engagement can also raise its own ethical issues [9]. For instance, engagement can create burdens on patient and family participants. Key justice-based issues relate to whether those engaged in research are truly “representative” of a broader patient population and whether marginalized groups are involved.

EOLPC research is arguably a unique research context [10, 11]. Patients may be vulnerable due to their state of illness or due to absent or intermittent decision-making capacity (e.g., in advanced dementia) [8]. Family caregivers may struggle to know and represent patients’ priorities or have difficulty engaging due to caregiver burdens or ongoing grief following a loss. The nature of EOLPC means that maintaining long-term relationships – often considered essential to meaningful engagement – can be challenging. Finally, any potential ethical challenges may be particularly relevant for individuals who are doubly vulnerable because of existing racial, ethnic, socioeconomic, and geographic disparities in access to palliative care [12].

Existing research, while recognizing these challenges, has not often explored how patients, families, and investigators experience the unique ethical challenges of engaging patients and families in EOLPC research. We conducted a qualitative study to obtain insights into the real-world challenges facing investigators, patients, and family caregivers in EOLPC research engagement and to use our findings to create practical ethics guidance for managing them.

## Methods

### Setting

This study was conducted within the Palliative Care Research Cooperative Group (PCRC). The PCRC is an interdisciplinary palliative care research community supported by the National Institute of Nursing Research of the National Institutes of Health. When the study was conducted, the PCRC included over 500 researchers at more than 180 sites.

### Sample

We recruited (i) investigators who had conducted patient- and family-engaged EOLPC research and (ii) patients and family caregivers who had been involved as partners or advisers in EOLPC research. For comparison purposes, we interviewed 6 investigators and 1 family caregiver who had not been involved in patient- and family-engaged EOLPC research.

To recruit investigators, we searched the PCORI database for funded studies related to EOLPC because these studies require patient and/or family engagement. This search identified 14 investigators, who were then contacted by a member of the study team (JK). Of these 14, 9 agreed to participate. We also recruited investigators via an email to PCRC members. Fifty-four people responded. To recruit among these 54, we used a purposive sampling strategy aimed at achieving diversity (“maximal variation” sampling) [13, 14]. Key measures of diversity for investigators included age, discipline, years in profession, race, ethnicity, prior experience with patient or caregiver engagement in research, geographic location, and practice site characteristics (i.e., urban versus rural; academic versus non-academic).

To recruit patients and family caregivers, we asked investigators whom we interviewed (as well as those whom we did not) to refer participants to us. We also asked investigators who expressed interest in our study, but whom we did not interview, to connect us with patients and family caregivers. For patients and family caregivers, we similarly recruited purposefully for diversity. Key measures of diversity for patients and family caregivers included age, race, ethnicity, socioeconomic status, and geographic characteristics (urban versus rural). Our final sample included 20 investigators, 19 caregivers, and 3 patients.

### Data collection

An interview guide was developed based on the study team’s knowledge of and experiences with ethical issues both in patient and caregiver engagement and EOLPC research. We modified the interview guide over time to allow insights gained from earlier interviews to inform later interviews. Semi-structured interviews were conducted over the phone by a member of the study team (MD or, in one case, AA) between August 2019 and May 2020.

### Data analysis

Our analysis employed grounded theory methodology [15]; specifically, Charmaz’s constructivist version which acknowledges that meaning can be influenced by the researcher’s own perceptions and interactions with the data and the participants [16]. After each interview, field notes and memos were created to describe emerging themes and patterns.

To create a preliminary codebook, two members of the study team (MD and AA) coded 5 investigator interviews and 5 patient/family caregiver interviews in order to create initial index codes and categories linking codes, as well as their definitions. During this “open coding,” the researchers met after coding each interview to discuss code interpretations.

Next, we uploaded transcripts into Atlas.ti (Version 8 Windows) to facilitate ongoing data analysis. We independently re-coded the first 10 interviews and half of the remaining interviews. Constant comparative techniques [17] were employed to clarify and refine codes, develop additional categories of codes, and postulate relationships between categories (“selective coding” in grounded theory). To help ensure intercoder reliability, the researchers both coded every fifth interview and met to reconcile coding results and discuss the codebook, modifying and re-organizing codes as needed. (The final codebook is available as Additional file 1). Through this process, we determined that thematic saturation had been reached after 35 interviews; we conducted 7 more interviews to confirm this.

The third and final step (or “theoretical coding”) involved several distinct analytic steps. First, we analytically linked challenges to ethics concepts, drawing primarily on foundational principles of biomedical ethics: respect for persons, non-maleficence, and justice [18]. As a matter of reflexivity, this choice was made partly because of the researchers’ own background in these principles, and the influence of these principles on research ethics, at least in the United States. Second, we identified those codes and categories most unique to the EOLPC setting, and identified a unifying theme (or “core variable”) from our analysis. Finally, we iteratively created several overarching ethics recommendations based on the data. The goal of this final step was more interpretive [16] than positivist [15]. That is, rather than create a single overarching theory of engagement with defined causal relationships, we sought to synthesize our findings, abstractly and conceptually, in relation to the unifying theme. The goal was to generate hypotheses, grounded in the data and related to the unifying theme, that can be refined and tested in future research. This allowed us to create practical ethics guidance tailored to the unique context of EOLPC research for engagement of patients and family caregivers that are directly related to the semi-structured interview findings.

Throughout we employed accepted methods to ensure analytic rigor, such as expert checking (e.g., by sharing draft findings and recommendations with EOLPC researchers at the PCRC annual investigator meeting in February 2020; our local palliative care research in progress conference; the American Society of Bioethics and Humanities Annual Meeting in October 2020; and University of Colorado Palliative Care Virtual Research Day in October 2020). To illustrate, it was the PCRC annual investigator meeting where it was suggested to create recommendations according to foundational bioethics principles with which researchers are already familiar, though as we discuss later our analysis found that this framing alone was inadequate. We also engaged in member checking (e.g., by sharing our draft findings, recommendations, and the codebook with research participants, including investigators and patients/family caregivers), reflexivity, and avoidance of selectivity in data use.

## Results

### Characteristics of participants

Each interview lasted approximately 1 h (range, 37 – 72 min) and was audio recorded and transcribed using a HIPAA-compliant transcription service. Demographic characteristics of our participants are in Tables 1 and 2. Our sampling of investigators achieved expected diversity in terms of age, medical specialty/area of palliative care, and method of engaging patients and family caregivers, but less diversity in terms of race (75% white) and practice setting (i.e., 3 non-academic sites). Our sampling of patients and family caregivers achieved diversity in terms of age, clinical condition, and average income, but less diversity in race/ethnicity and gender. These patients and family caregivers were mostly involved in patient advisory boards or multi-stakeholder boards that could include clinicians and other stakeholder types.

**Table 1:** Demographic characteristics of interviewed investigators (*n* = 20)

Characteristic	N
**Age**	
30-39	5
40-49	10
50-59	3
60-69	2
**Gender**	
Female	14
Male	6
**Specialty/Discipline**	
Palliative Care	4
Oncology	3
Nursing	2
Internal Medicine	2
Other	9^a^
**Race/Ethnicity**	
White (non-Hispanic)	15
Asian American	3
African American	1
More than one race	1
**Method of Engagement**	
Multi-Stakeholder Advisory Board	7
Patient/Family Advisory Board	5
Patient Co-Investigator	2
No Prior Engagement	6
**Practice Site**	
Academic	17
Non-academic	3

^a^One each from Anesthesiology, Neurology, Emergency Medicine, Pediatrics, Psychiatry, Nephrology, Geriatrics, Anthropology, and Social Work

**Table 2:** Demographic characteristics of interviewed patients and family members (*n* = 22)

Characteristic	N
**Age**	
20-29	1
30-49	1
50-59	8
60-69	2
> 69	10
**Gender**	
Female	18
Male	4
**Clinical Condition**	
Cancer	12
Neurological Disease	5
Cardiovascular Disease	2
Kidney Disease	2
Liver Disease	1
**Race/Ethnicity**	
White (non-Hispanic)	18
African American	2
Hispanic	1
Unspecified minority	1
**Method of Engagement**	
Patient/Family Advisory Board	11
Multi-Stakeholder Advisory Board	10
No Prior Engagement	1
**Average Annual Income**	
< $50,000	9
$50,000-$100,000	7
> $100,000	6
**Rural/Urban**	
Urban	19
Rural	3

### Engagement challenges and their management strategies

Table 3 displays ethics challenges and potential solutions found in our interviews. For brevity, we present only those challenges we deemed as relatively unique to the EOLPC setting. For example, interviewee-reported challenges related to needing more time, more funding, or the general funding structure of research (which some participants thought might limit patient/family input from the outset) are not included unless they brought up issues unique to EOLPC (e.g., additional time needed to engage with people who have neurodegenerative or cognitive decline). However, a full table of all challenges and potential solutions is available in Additional file 2.

**Table 3:** Challenges and potential solutions identified in interviews (Numbers correspond to instances of coding; I = investigator interviews; P&FC = patient and family caregiver interviews)

Challenges	Codes present			Potential solutions	Codes present		
	I	P&FC	Total		I	P&FC	Total
**Facilitating participation of those with serious illness**	29	12	41	**Have meetings in the community and/or hosted by trusted community organization - at least initially.**	1	2	3
				**Use virtual methods to overcome challenges (such as travel)**	2	10	12
**Managing issues of death and illness, including progression**	23	8	31	**Engage patients before they are too sick**	1	1	2
**Avoiding paternalism or gatekeeping**	12	5	17	**Recruit sensitively and unobtrusively**	5	9	14
**Minimizing burdens**	5	10	15	**Have support services available (e.g., parking)**	2	2	4
**Navigating decision capacity or its fluctuation**	7	2	9	**Have family caregivers involved to support cognitive limitations**	3	1	4
**Dealing with different communication abilities (e.g., due to neurological illness)**	3	4	7	**Allow additional time for communication**	1	0	1
				**Have short or alternative means of feedback**	7	11	18
				**Have a peer partner to support the participant**	3	1	4
**Finding the right time to recruit partners, including caregivers**	2	4	6	**Recruit sensitively and unobtrusively**	5	9	14
**How to respond when the patient and caregiver voices are conflicting**	3	0	3	**Having a joint patient and caregiver together on the team still creates useful dialogue**	2	1	3
**Totals**	84	45	129	**Totals**	32	47	79

We initially attempted to distinguish “ethical” challenges from “other,” more logistical challenges. However, we found in particular that patients and family caregivers responded to our questions about “ethics” challenges differently than investigators. Although investigators were comfortable speaking about traditionally taught research ethics principles of respect for autonomy, beneficence, and justice, patients and family members did not see ethics only through these lenses. Recognizing that almost any challenge has an ethical dimension -- i.e., what might seem a simply matter of logistics, such as meeting time or day, can relate to ethics principles of beneficence (by minimizing burdens) or justice (by promoting equal access) -- we collapsed all challenges into one category. Doing so meant we did not privilege ours, or our investigators’, definition of ethics in our analysis.

### Authenticity as a unifying theme

During our analysis, “authenticity” – understood as describing an engagement activity that is motivated by an honest desire to listen to the voices of patients and caregivers and that includes specific practical actions that make that possible – emerged as a unifying theme. Authenticity appeared explicitly in some of the data – we used the code “being authentic about engagement” to capture instances of this – and through our analysis, we found that many codes reflected authenticity as well. Examples included “being explicit that the researcher will listen first,” “engagement changed the course of the research,” and participants who “felt valued.” By connecting these codes to authenticity, we found that this theme was present in almost all of our interviews. In addition, not only did we capture specific codes around authenticity in 35 of 42 interviews, but also it became clear throughout our analysis that many of the challenges in Table 3 were meaningful to patients, families, and investigators because of how these discrete issues and actions in response reflect authenticity. Likewise, ways of overcoming challenges could be seen as promoting authenticity.

To illustrate, participants described how alternative forms for feedback (outside typical board meetings) can demonstrate the sincere desire to receive feedback from patients and family caregivers:

“So having the right people in the room and making sure that the questions that are asked are genuine and actionable, I think is really important. So I think that's probably the biggest ethical concern that I have in putting CABs together, is that it's not-- I'm not checking a box. It's not so I can say, ‘Oh. I did this in the culturally right way because boom I had a CAB [Community Advisory Board].’ Tick. I checked that box off. It is about truly getting the input that you need in order to ensure that what comes out of an intervention development study, if that's what you're doing, or out of a trial, or out of whatever, is in fact something that can be made actionable...” [Investigator, Female, White, Age 49]

Authenticity, therefore, as a concept represents what we took to be the goal of engagement, particularly for patients and family caregivers.

### Ethics recommendations for authentic engagement

In the final, most abstract phase of our analysis, we synthesized our analytic findings by creating recommendations as the theoretical output of our constructivist approach (see Table 4). These four distinct recommendations were meant to link the challenges we observed (see Table 3) with the concept of authenticity; they can be seen as hypotheses generated from our data for how to improve authentic engagement in EOLPC research. In this section we expand on the basis and implications of each.

**Table 4:** Proposed recommendations for engaging patients and family caregivers in end-of-life and palliative care research

Recommendations	
**1. Give patients and families the opportunity to participate as research partners – even very near life’s end - and do so sensitively and with compassion.** For example, patients and caregivers appear willing to be asked to partner, even close to life’s final days – potentially providing crucial, untapped insights into the design of patient- and family-centered palliative care.	
**2. Proactively minimize the burdens of patients & families participating as research partners by being thoughtful about when, where and how engagement activities occur.** For example, to minimize burdens associated with serious illness, investigators can propose short feedback sessions at times and places convenient to patients (e.g., feedback sessions at the infusion center, rather than a traditional evening board meeting).	
**3. Take steps to increase diversity – broadly construed – in engagement partners.** Diversity comes in different forms; some patients and families reported a need to engage diverse races, ethnicities, and even more individuals who identify as men (recognizing that most family caregivers identify as women).	
**4. Be prepared to manage “relationship ethics” as a result of the deep bonds that can form over time with partners**. Some issues that arise – such as blurred boundaries between investigators and patient/family partners or even the rare need to end a research partnership – do not fall neatly into traditional research ethics principles (and these issues require further research).	

Recommendation 1, based in respect for persons and their autonomy, reflected our participants’ strong belief that patients and families should decide for themselves whether to engage. Just as many reject the idea of “gatekeeping” in EOLPC research – i.e., when clinicians or others prevent or withhold research opportunities from potential research participations [19–21] - so too was the idea of “gatekeeping” rejected when it came to acting as a research advisor or partner. As one participant said:

“I think that’s part of that paternalism that I was talking about, when we want to protect others from what we might anticipate as harm. That can be reframed into the opportunity to have the autonomy to choose, can be seen as a gift, so the person can look you in the eye and say, are you crazy?... But I get the choice. You didn’t preselect me out because of my end-of-life status, or my symptom-burden status, or my whatever.” [Investigator, Female, White, Age 61]

To reject gatekeeping also requires taking steps to allow individuals with reduced decision-making capacity to participate by empowering them to do so (e.g., by pairing them with a caregiver or advocate, or allowing them to provide feedback in ways outside of a meeting or conference setting).

Practically, our patient and family participants (more so than our investigators) were comfortable being approached to participate even very near life’s end. This is a critical insight. If researchers believe that patients and family caregivers ought not be approached as research advisors very near the end of life or shortly after a loved one passes away, this could inadvertently create a crucial gap in the knowledge base of EOLPC – one that patients and families want to fill:

“I guess, I mean, I know if I was going through it, like I'd want to have my opinions heard. If there was ever a time to get your opinions heard, it's the end of life. So as long as I didn't feel like I was being exploited, and it was my decision to be there, then yeah, I would want the ability to be there.” [Caregiver, Female, White, Age 31]

“We want to contribute. We want to give something back. And, I mean, I have to say-- again, I have to use my mom as an example. My mother would have been more than willing to give her opinion about anything, end-of-life care, or just caring for the elderly, period. And I know that end-of-life care doesn't just include elderly people, but my mother would have been willing to give her opinion or herself in any way if she thought she was leaving something better behind. So I think it's a difficult question to ask, but I think it's also an important question to ask because you won't know until you ask.” [Caregiver, Female, Black, Age 55]

Doing so sensitively and with compassion still implied the need to do so carefully. Concretely, before asking about research engagement, it may be important for the research team to be upfront, asking openly and honestly about whether it is a convenient time to ask about research engagement and ensuring there are no competing personal or social needs that should take priority. Some, but not all, family caregivers were comfortable being recruited as advisors even within a month after a loved one’s passing – and none felt that having a known clinician do the recruiting exerted inappropriate influence. Similarly, researchers should be aware of potential cultural differences in the meaning of death and dying (e.g., in some cultures, it is taboo to talk about death or the deceased by name) while not stereotyping by merely assuming such differences exist.

Recommendation 2, based in obligations of beneficence and non-maleficence, reflected the idea that EOLPC research – by being frequently situated in settings of serious, chronic, or life-limiting illness – can require additional efforts (when compared to traditional research) to minimize the burdens of participating in research engagement. Arguably a dominant model in contemporary research is the advisory board – a group of patients, family caregivers, and others who meet regularly to provide input to a study. Whether this model serves EOLPC research depends on the details of a particular study; however, there is a need to be particularly considerate of burdens posed by meeting time, location, and especially the built environment (e.g., around access for people who are variably abled). And in the setting of EOLPC, researchers should be mindful of the potential harm caused by evoking difficult emotions and grief when discussing end-of-life issues.

Researchers also should be willing to obtain input in ways outside the traditional board meeting setting by meeting patients and families where they are in the hospital, the emergency room, the dialysis unit, or even at home (assuming that the participant is willing and able to engage comfortably in those settings). In addition, being accepting of short feedback sessions, done more frequently, can be less burdensome than meetings lasting over an hour. As one participant said:

“To have any consistency, they have to make multiple quarterly meetings. And that’s just not something that people with serious illness are often up for. And I think that’s important because then there’s an important voice. Family members provide a great perspective, but there’s an important voice missing if the patients aren’t there. And that’s why we go to one on one interviews sometimes by telephone or we travel to patients to make sure that we get that perspective as well.” [Investigator, Male, White, Age 59]

Caregiving can create additional unique or unrecognized burdens. When caregiving demands prevent participation in meetings or conference calls, researchers should plan for and be prepared to follow up separately if needed to ensure that the patient and caregiver voice remains heard. As a result of the COVID-19 pandemic, virtual methods of engagement are of particular interest. These methods can minimize travel and time burdens while still approximating face-to-face interactions. When used, however, there must be attention to inequities in technology access and technology literacy:

“One of the things that we found was that it was hard for patients sometimes to participate depending on the stage of the disease that they were in. Even if we didn’t ask people to come, like we all try to meet at the campus. But we also offer the possibility of joining by Zoom or by telephone. So even at that, for Parkinson’s patients, sometimes communication is a problem.” [Caregiver of a patient with Parkinson’s, Female, White, Age 72]

Recommendation 3, based on obligations of justice and fairness, arose because of diversity concerns. While diversity concerns are not unique to EOLPC, they were the most frequent ones we encountered in interviews. Concerns about diversity and inclusivity have been repeatedly expressed regarding research participation and regarding participation in research engagement generally [22–25]. Diversity in terms of race, ethnicity, and socioeconomic status, among others, were identified as key challenges for EOLPC research. This may be reflective of broader disparities in access to palliative and hospice care – disparities that are compounded when it comes to engagement [26]. For instance, individuals who are well off or who do not work may find it easier to participate (or be more likely to be invited).

In addition, interviewees noted some unique diversity challenges in end-of-life and palliative care, such as the need to include more family caregivers and more male voices (e.g., because family caregivers tend to be female). The following two quotes are illustrative of concerns around justice.

“Well, one thing I wanted to mention is I think we’re one of the very few Spanish speaking councils in the nation…I think it would be great for every hospital to have a Spanish speaking council or a Chinese speaking council, and any other language council because our needs are very different. Every culture and language has their own specific needs.” [Caregiver of a patient with cancer, Female, Hispanic/Latina, Age 29]

“A lot of careers that men get into don’t afford them the opportunity to attend, especially when they’re the primary caregiver or just caregiver of a family. They provide the resources for their family. But we definitely need them at the table because we need them to go out to share this information with people who look like them regardless of whatever their ethnicity is. They need to be able to go back into their community as men and educate the men about how important it is for them to do these things.” [Caregiver, Female, Black, Age 64, End-stage Renal Disease]

Practically, this means actively working to involve ever more diverse engagement partners and to ensure diversity is broadly construed. Building on Recommendation 2, participants noted a need to make special efforts to minimize burdens of participation that may make it hard for certain individuals to participate. For instance, the burden of meeting during daytime working hours can be disproportionately borne by individuals who work in certain jobs or vocations; as such, minimizing burdens by holding evening or weekend meetings (which may be less convenient for the research team) can simultaneously encourage more diverse participation.

Recommendation 4 arose based on our analysis of issues and challenges that did not fit neatly into the three aforementioned principles of bioethics. As a result of widespread educational requirement, researchers (but not necessarily patients and family caregivers) are steeped in human subject protections and these principles. However, the traditional research ethics model has limitations. It tends to view human subjects as being uniquely vulnerable and requiring special protection (even if recent efforts have tried to move this model toward fair participation, not just protection). Yet patient or family research advisors, partners, and co-investigators do not clearly fit into a position of vulnerability, at least not in the same way, and many believe the connotation of partner to be more indicative of equals. Moreover, the motivations for participating as a research partner may be different than those for enrolling in research, and these different motivations may affect relationships. Although specific motivations did not arise in our research interviews, both investigators and patients or caregivers might do well to be upfront about these as part of relationship building.

If the research ethics model is a poor fit, many additional and intriguing ethical questions arise. When is it appropriate to end a partnership with an advisor, and how should this be done? How should a researcher respond if a patient or family caregiver partner asks for medical advice or other support? Are research partners “friends”? Is it appropriate for researchers and partners to be “friends” or to interact socially? Below are a few examples of these sorts of situations:

“She was a partner and she’d been on this journey with us for a year and change. And I knew she liked visits and she didn’t have a lot of family…And so we got to know her friends, and her friends said they’d love us to visit. And so I remember I brought my sons and my husband to spend an hour drive away, and my son had colored a whole bunch of things that we could decorate her hospice room.” [Investigator, Female, White, Age 42]

“She is one of the most dearest friends in my life. She is remarkable. She is wonderful. She’s compassionate, she’s sensitive, she’s smart. And she used to always tell me – what I loved most about her, she said, ‘I’ll do the research part. I’ll do the writing part. You just be you.’” [Caregiver of a patient with end-stage renal disease, Female, Black, Age 64]

“We had what I call a non-normal relationship…in the sense that it really was much broader than a patient-doctor relationship. There was really an interest in each other as human beings and people, and we just so respected his commitment to the patients and families.” [Caregiver of a patient with Parkinson’s, Female, White, Age 71]

“So that’s kind of outside of that whole patient advisory role because these are human beings, you know? And they bring all of their – like you’re in a – we were in a relationship now, just like any working relationship.” [Investigator, Female, White, Age 46]

“And I really had a nice relationship, fun relationship with the principal investigator. And she is the one that I still work with on this other study they’re now doing. So I have kind of an ongoing connection from that. [Patient with cancer, Male, White, Age 75]

Understanding and addressing the potential for blurred boundaries and managing expectations upfront become key.

## Discussion

Our study adds to a growing body of literature around engaging patients and families in EOLPC research [9, 27–33]. A systematic review, published near the end of our data analysis, identified 25 published articles reporting on ethical considerations in engaging frail and seriously ill patients as research partners; issues identified by content analysis were, like our findings, grouped into traditional principles of biomedical ethics [33]. In this section, we highlight what our findings add to this body of literature and further expand on our qualitative analysis.

First, our study further demonstrates the value of directly asking patients and families about ethics challenges [9], rather than relying solely on researchers’ beliefs or reviews. In our study, we found that new and concrete management strategies for ethical challenges came more often from patients and families (see Additional file 2).

Two additional observations from our findings shed light on the importance of elevating the patient and family voice. First, when analyzing the views of investigators who had not yet engaged with patients or families as research partners, we found that inexperienced investigators expected challenges (e.g., around overly burdening families simply by asking, about not approaching them directly, and so on) that our patients and family caregivers found insignificant. Our findings and recommendations should reassure researchers and (contra “gatekeeping”) provide encouragement to engage patients and families more, not less. Second, we had a strong sense from the experience of conducting our interviews that it was difficult to get patients or families to express some issues, particularly burdens of engagement. Many seemed simply thrilled to participate in engagement and required direct prompting to endorse even simple burdens, such as parking or disability access. In practice, implementing Recommendation 2 (minimizing burdens) will therefore require careful and proactive efforts to elicit unintended or unforeseen burdens of participation. Regular evaluation of engagement activities [34–36] that include assessment of burdens could be a practical way to lower the activation energy that might be required for patients and families to bring these up on their own.

We observed important differences in language around ethics (hence our inability to delineate clearly “ethics” challenges from “other” challenges). From this finding, we conclude, for example, that there is not necessarily a need to teach patients and family research partners the classical principles of biomedical ethics for them to be able to think and talk about ethics with researchers. Doing so might limit the moral imagination and analysis necessary for identifying new issues or ways of managing them. There appears to be a need to find common ground and language while preserving the unique voices of patients and families.

To illustrate this, and similar to a recent review of ethical issues in engagement in research generally [37], we found a category of issues not captured by classical research ethics principles: relational ethics. In Recommendation 4, we note that researchers must be prepared and aware that patient and family engagement in research may require a different approach than a traditional researcher-human subject relationship that is based on protection or even vulnerability. From an ideal perspective, some see the goal of engagement to create relationships of equals or near equals (hence language such as “partner”). This suggests a need to consider analyzing ethical issues of research engagement through an additional lens of relational ethics or the ethics of care [38, 39]. Such an approach makes concepts such as trust and relationships among people the fundamental units of moral analysis, rather than discrete, autonomous agents and principles [40]. The implications of such a paradigm shift should be explored in future studies.

Authenticity has recently received greater attention as an important concept in research engagement [41]. Although defined by some as ensuring patients and families are full partners in the research, in our view, authenticity applies no matter how deeply a patient or family caregiver chooses to be engaged in EOLPC research. It describes a genuine, honest, and openly transparent desire to take seriously the advice of patients and families and to treat them accordingly with respect, care, and concern. Authenticity is not unique to EOLPC research engagement, but our findings suggest that the EOLPC research context has unique characteristics that must be acknowledged to support authentic engagement. Authenticity in EOLPC research requires attention to issues even very near life’s end, recognizing the unique burdens of engagement in EOLPC and taking steps to minimize them, promoting diversity, and considering the unique relationships forged out of EOLPC. Future research studies should test whether and/or how the recommendations from our study promote authenticity in EOLPC research.

Like all studies, ours has limitations. As a qualitative study, we are unable to make claims about the population-wide frequency or importance of particular ethical challenges. In addition, qualitative research involves subjectivity in analysis; member checking and reflexivity can minimize, but not eliminate, this. Lastly, despite our attempts to recruit for diversity within a large, multidisciplinary research collaborative, our findings may not generalize beyond our participants.

## Conclusions

Our study has provided four practical recommendations for ethically engaging patients and families in EOLPC research based upon the concept of authentic engagement and focusing on those issues most unique to the EOLPC setting. These recommendations stand together, not alone: Minimizing burdens (Recommendation 2) unique to certain patients and families can be a way to improved diversity (Recommendation 3), for example, and researchers ought not approach patients in the most convenient place for the sake of minimizing burdens if doing so unduly influences someone to participate (e.g., if an individual feels they have no choice but to participate while in the hospital). While we wholeheartedly endorse other recommendations common to all research (e.g., around compensating participants, providing appropriate training and resources, maintaining privacy and confidentiality, and so on), there is added value in recommendations specifically tailored to the EOLPC context.

## Supplementary Information

**Additional file 1.**

**Additional file 2.**

## Data Availability

The datasets used and/or analysed during the current study are available from the corresponding author on reasonable request and will be available from the Palliative Care Research Cooperative De-identified Data Repository https://palliativecareresearch.org/studies.

## Abbreviations

EOLPC: End of life and palliative care
PCORI: Patient-Centered Outcomes Research Institute
PCRC: Palliative Care Research Cooperative
