# Supplementary material for: Meeting ethical challenges with authenticity when engaging patients and families in end-of-life and palliative care research: a qualitative study
Source: BMC Palliat Care. 2022 May 16;21:74. doi: 10.1186/s12904-022-00964-x (PMC9108140; doi:10.1186/s12904-022-00964-x)
Supplement: Supplementary file 1 — Additional file 1. [file 12904_2022_964_MOESM1_ESM.pdf]

Appendix 1. Codebook

CATEGORIES	CODES	MEANING	EXAMPLE QUOTES
**DISCUSSING WHETHER PALLIATIVE CARE IS UNIQUE**	Used when the interviewee explains what, if anything, makes EOLPC research unique compared to other medical settings		
	"Sensitive" issue, but people want to talk about it.	EOLPC can be a sensitive or emotional topic, but people actually do want or need to discuss it	"I think a lot of-- I mean, I'm just thinking from my own personal experience. I think that experiences that I have had, are what make me feel that I really want to make a difference. And I think that a lot of the patients and caregivers that participate, have maybe had similar experiences. So though that can be a challenging topic, it's also an important topic."
	Intensifies ethics issues	The setting of EOLPC makes ethics issues more important or more pressing	"So you're right. I think all of these have implications for all research but again, because palliative care just heightens the potential for all of these things to be kind of on steroids, right? But the idea that this just ups the ante on all of this, again, there's more likelihood that the person who is living the experience of meeting eligibility criteria for palliative care, which in today's world, again, does mean serious illness and often, again, pretty high symptom burden and possibility of, again, being close to end of life, all of those just compound all the things that we're talking about just all the more so."
**DESCRIBING KEYS TO SUCCESS**	Used when the interviewee describes the most important factor that improves engagement		
	Success defined by the group	The patient/family advisor or group should define its own success and not have a definition imposed on them	"But the idea that we are, in fact, serving a community is something that I think is very-- or serving a population, and that the work that we do must be mutually beneficial, at the very least, if not way in the direction of the population or the community, from their perspective, not from what I define as being to their benefit, but what they define as being of interest and to their benefit."

	Being explicit that the researcher will listen first	The researcher must be ready to listen as part of open communication	"I think first of all would be don't have any preconceived notions. It's good science to let the evidence drive you and patients. That means listening to them first and, plus, about asking them questions. Sometimes if you don't ask them a question, they're going to tell you something you didn't even think about. So I think that's probably the most important thing."
**EXPLAINING 'WHY' DO ENGAGEMENT**	Used when the interviewee explains the 'why' of having patient engagement, whether via a board, advisor, partner, etc.		
	To tailor interventions to the community	Engagement helps ensure the intervention tested is relevant to and respective of the community, its cultural norms, etc.	"So there are all these religious factors, there are cultural factors, about how people react to medicine, how they talk to doctors, how their families are willing, whether their families are willing, maybe their families aren't willing, and it could just be their cultural reasons why they're hesitant or reticent for doing all this, and the medical world has to understand that. Otherwise, you're not treating the whole human being; you're just-- just because something is right for a 70 -year -old white Christian, doesn't mean it's going to be right for a 35 -year -old Black Islamic. I mean, it's just one size doesn't fit all. And I'm just hoping that this movement to get patients more involved, and their families more involved, continues to morph and grow."
	To improve dissemination and implementation being community driven	Engagement improves dissemination and implementation because something done by the community is more likely to have uptake	"And then at the end of the study, we did reconvene the group in person, go over the results. And we'd sort of brainstorm together what venues we would want to try to share the information and disseminate the findings more broadly. So they were part of that final stakeholder meeting as well."
	To comfort the researcher because it means the researcher is not "in it alone" regarding research	Engagement provides a way to share the burden or the accountability surrounding details of the project in a way that comforts the researcher	"One of the things that I-- I think the thing I most liked about that advisory board and the advisory board I work with, is the assurance that it provides me, especially as a non-native researcher and not a member of a community

			where I'm doing the research, it provides me with a great deal of assurance that we are "getting it right," that we are designing an intervention or developing an intervention or tailoring an intervention that will, number one, be of interest to the community, that's addressing a problem that's important to the community, that, number two, will be acceptable at the very least that will be acceptable to the community and appropriate in what it looks and feels like to people using it, and at the very most will be beneficial to the people, and will actually improve health and well-being of patients and family members, and hopefully also people working within the healthcare system."
	To foster trust/rapport in the research enterprise – particularly for communities with historical distrust	Engagement can improve trust in the research and the researcher in ways that can support other aims (e.g., recruitment; see below)	"I felt we would get nowhere without buy-in from both providers and family, which is why I've felt that engaging family one reason this research is really important because it's one thing to be able to, in a bubble, say that this is the way we can best take care of our patients and advance the research that we're doing. But without the engagement of the stakeholders, the very important stakeholders, and especially it's a very emotional and high stakes period of time in any person's life and the life of a loved one. And I don't see how we're going to be able to do this kind of work without involving the perspective of family members and/or patients."
	To improve the quality of research in a general, non-specific way	Engagement is a way to improve the quality of research in general, such as regarding the outcomes chosen, its conduct, etc.	"It went very well. I mean, the patients were very engaged and provided a lot of good feedback, and, yeah, I think it kind of validated our idea that you really don't know how people feel about things till you ask them, which is kind of obvious but novel for doctors."
	To get feedback on a discrete research-related item	Engagement provides a way to get feedback on a discrete item, such as a consent form or documents, as part of the research	"But when we go through everything we'll talk about now we need to concentrate on patients and caregivers. What do you need to see in a

			program? What do you feel is important that we learn? And even when it comes down to looking at reports, surveys, forms that they're having everyone fill out, the patients and caregivers get the opportunity to look at those, and we can say, ‘Okay, you need to dumb this down a little bit. Or how about you phrase it this way, it's not so cold and harsh.’”
**DESCRIBING IMPACT OF ENGAGEMENT ON RESEARCH**	Used when the interviewee explains something tangible that happened to the research		
	Engagement changed the course of the research	Having patients/families involved resulted in a change to how the research was conducted (e.g., what outcome was evaluated, what questions asked, etc.)	“So I think she brought so much to the role, and, specifically, she felt strongly that loneliness was an important outcome for our palliative care study. And she really advocated, quite convincingly, that we needed to add that as an exploratory outcome because it's of primary importance to people towards the end of life. So we changed our whole protocol, and added it.”
	Patients and/or family members participated in the data analysis or its writeup	Patients/families were involved in the analysis or publication in ways that improved the research	“After that, I think most of our participation came when we were doing up the qualitative part of the study. They'd give interviews just like you're doing. And so they would send us a few transcripts. I've forgotten how many we did. Well, a total of 60 interviews were done 30 patients, 30 care partners. But every month or so they would send us maybe four or five transcripts that they wanted us to look over. And then we would meet and talk about what we saw as some of the most important parts that we picked up from the interviews.”
**DESCRIBING THE MAKE-UP OF THE BOARD or GROUP**	Used when the interviewee describes the characteristics of a patient/family board (i.e., when boards were the method of engagement)		
	Primarily members of the community	The board was a mix of community members (though others may be included)	“And so, like a lot of the studies we do, we formed a Community Advisory Board that was made up of Native American people and Alaska Native people from our two sites. So we had six

			community members, some of whom are also healthcare professionals, or otherwise involved in the healthcare system in their respective setting. But all of whom were members in some capacity, either by marriage or by being born into the Alaska Native or American Indian communities where they live.”
	Included national representation	The board was meant to be representative at the national level, not just an individual community	“No, not just-- mostly not patients. Mostly people from well-known national organizations for palliative care, I think, or doctors.”
	“Diverse”	The concept of “diversity,” broadly construed ,describes the board	“Diverse in professions, education, race, even, probably, some cultural beliefs, in the sexes as male and females. There were whites and blacks and maybe more Native American than the rest of us, so Native-American guys, Asians, and Spanish. So there was a huge-- I mean, there's a lot of diversity from the ground up. Some people had only finished high school. Someone hadn't finished high school, some people that completed college. Some people are doctors. Some people have master's degrees. Some people had PhDs. So it was a great diverse group.”
	Having a patient advocate chair	The board is chaired by a patient or patient advocate	“Yeah, we developed a panel, and we got somebody who was involved as a patient advocate here at the hospital. She kind of chaired it. And then we got patients and family members, dialysis patients, transplant patients, family members all involved.”
	Included few patients and caregivers	The board is made up of a relatively small number of patients and caregivers	“It's just like they didn't involve people the way they could have. I mean, the doctors didn't want to refer patients to palliative care. The patients didn't want to hear about palliative care. Well, that's a block. And how do you get through that? And I don't think enough people of the patients were involved or even core doctors in designing the whole idea creatively.”
**DESCRIBING GROUP DYNAMICS**	Used when the interviewee describes how the group works together, as a whole		

	Mutual respect	The group members respect each other	“Yes, without a doubt. That was one of the fun parts about going to the meetings because then you get to see people. And you get to see that people really appreciated, at that point, the fact that you were putting input in. Where it went, I don't know. But I didn't ever feel like there was any frustration or animosity.”
	Common goals	The group shares common goals	“Because I think the people who were part of it, number one, had a passion for the field. So they had a-- they brought their passion with them. And I think that was because of what their day to day responsibilities and their careers represented. And so I think that is where the respect came. Everybody brought something to the table. There was no big yous, little yous, big I’s. We were all a team working together because at the end of the day, we all understood that, whatever the outcome of our research, was going to impact thousands and thousands of people.”
	Challenges can come with a larger group	When a group is large, it can bring challenges in managing dynamics	“So with the big, big meetings, it can be a little overwhelming sometimes just because I don't understand a lot of it. The smaller meetings, that's when we can get really nitty gritty into how we see things, what we want to see in maybe a palliative care video, items on that order.”
	Managing a strong personality	Group dynamics can be affected by a strong personality (including when it is an administrative or support person)	“I mean, I think she probably would've been somebody, had we continued and had she continued with that energy, that we would have either have to take her aside and talk to, to focus, or possibly even excluded from the group. And the thing from talking to other people that she was a bit disruptive for support groups that she was a part of but basically just decided that she didn't want anything to do it. Which was fine with us.”
	Importance of a good leader	Having a strong leader is an important way to manage group dynamics	“We've been fortunate about that. Yeah. And actually even within the group I think people-- again, they just kind of defer to [name] and

			everyone has a lot of respect for him and for his leadership, and he has a lot of integrity. So he was a great person as well as [name]. I mean, they're just outstanding individuals.”
**DESCRIBING OTHER ENGAGEMENT ACTIVITIES**	Used when the interviewee describes other “engagement” activities, either beyond the formal ones (informal advising) or in the use of “direct” surveys or focus groups		
**DESCRIBING HOW TO IDENTIFY ADVISORY BOARD MEMBERS**	Used when the interviewee describes how research engagement partners, Co-Is, board members, and/or patient/family advisors were identified		
	Because of their knowledge	An individual was chosen because of knowledge of the subject - either specifically or in general	“Yeah. Well, she was a dialysis patient, very high up in a large organization, a renal advocacy organization, and so not only had-- let me just silence my computer. Not only had she had a lot of personal experience, but had advocacy experience on the national level, and so understood sort of more broadly what other people's experience was.”
	Because of their status as a key decision-maker	An individual was chosen because of some power/authority/influence to make change or get desired buy-in	“We need somebody who has enough authority within the community and/or the healthcare system, whatever drives what in that community, to actually help us make decisions, but get us closer to that or help make that possible.”
	By patients or families themselves, i.e., when they identify others	It was important for patients/families already involved to identify members - not just the researchers	“I think it was about-- I think it was about five. I think we offered it to the patients that we knew that we were caring for. And I think the patient advocate also reached out to some people that she knew.”
	By working with partners or organizations	Engagement participants were identified partner organizations (e.g., via advocacy groups, others)	“And so I have some really good relationships with the [name of organization] and some groups here in [geographic location] that focus in those diseases, gynecological cancers more broadly, and build up relationships with other just health-related groups that focus on patients that have some type of vulnerability whether it be because of where they live, because of their

			socioeconomic status, whatever. And because I've spent that time, and it really does take a lot of time to build up that trust in the community and amongst people whose jobs and missions usually as nonprofits are to protect and enhance the lives of these individuals, that I am able to work with them so well and they know me. They trust me when I am looking for whether it's patients or caregivers to be involved in my work.”
	Because it was clear the patient/family caregiver could go beyond their own story	An individual was chosen because of the ability to get beyond their own personal experiences, and to think about patients and families more generally	“But I guess also because I think in an advisory board, you're sort of asking them to step back and take a look at a little bit more globally at things I think. And I guess in my experience, I have yet to meet somebody in that first week with such a huge-- in hospice anyway, not palliative care, but in hospice have-- would have capacity to kind of step out to be able to look and answer broader questions. I don't know, not because they aren't capable. But rightfully so, they're incredibly focused as big transition of care. But I guess immediately after that in the recent months, I think we would find a few individuals who had that capacity. “
	Because it was a patient or family caregiver clinicians knew	An individual was chosen because of an existing relationship with a clinician (of any duration)	“We really just chose people that many of us had worked with clinically in the past that were very much interested in our research and had supported our research in other ways.”
	Because the patient/family caregiver had experience as a target of the study’s subject matter	An individual was chosen because of relevant experiences that would make them a subject of the study (as distinct from knowledge of the condition)	“And then we also wanted people who had patient perspective to offer. So one of the people-- actually, two of the people we initially invited were members of the community who-- one who had been through treatment for cancer, for a potentially life-threatening cancer, and one who was in-- had late-stage cancer at a time.”
	Because of the patient’s/family caregiver’s involvement in an existing	An individual was chosen because of already being involved in a PFAC committee at the organization	“And I went to the patient and family advisory council because my collaborator mentor had said, "If you want to get a patient advocate and maybe start talking to the PFAC." And I went to the PFAC

	patient and family advisory council (PFAC)		and presented this study and said, "I'd really love to have somebody work with me, a patient or family member work with me on this study just to start processing the data and to talk through things and what we are finding and such."
	Because of the need to include diverse members in the engagement	An individual was chosen specifically to meet diversity needs	"I think as we were going through our data and doing kind of a preliminary analysis that our sample and our final sample really is not very diverse. Like most studies, it's mostly white people who are highly educated. And so I had a patient who was African-American who was running a support group who is actually-- I have spoken to before. I think actually I spoke at her support group and she was really interested in how to get African-Americans and Hispanic people with Parkinson's more engaged and to get them more active about taking care from movement disorder specialists and going to the university and things like that, so that was a pretty natural fit and fit a need for the study."
	Because the patient/family caregiver is able to work well with others	An individual was chosen because the investigator was able to tell that they could work well with other members of the team	"Well, if was doing other studies in other areas I would choose patients in a different way. And I guess, part of it is just kind of a gut feeling of how well they're going to work with others."
**DESCRIBING HOW THE P/F RESPONDED TO BEING RECRUITED**	Used when the interviewee reports how they felt when being recruited to be an engagement partner		
	Honored	The interviewee expresses a sense of being honored or flattered at being asked	"When the opportunity came available to participate in the research I was honored to have been asked and because I'd worked very closely with the advisors of the lay health education program and they knew my passion and they also knew this researcher who had this passion and it just seemed like it was just a good fit. That's exactly what it was."
	Wanted to advocate	The interviewee expresses a desire to use the invitation as a way to advocate	"And when I found about the council, I decided it was time, that it was a good opportunity for me

			to join and make a difference for those that don't have family members that can help them out during that difficult time in their lives.”
	Being OK with care provider recruiting	The interviewee expresses the idea that it is acceptable for a care provider of the patient to do the recruiting	“I would say I don't-- I would have respected my doctor's input and the fact that he felt like we would be good participants in the study. But I can't say that it would have been any different than the person that contacted me.”
	Feeling pressured to participate	The interviewee expresses the idea that there could be pressure to participate	“I mean, I guess I'd always be concerned that you're somehow going to receive either favorable or unfavorable treatment of patients if your patient's still alive. My grandmother passed by the time it started, but I also didn't really consider it when I signed up for there to be some type of ramifications for her care, because I was part of the study. Yeah, I guess that would be the main thing someone could be concerned about.”
	Excited	The interviewee expresses a sense of excitement at being asked and having the potential to participate	“Yeah. I was excited. I was really excited first of all to know that he was doing that. He's such a progressive guy. But just to have an opportunity to kind of be at least on the edge of research again because by that time I'd been retired for close to 10 years. So yeah, it was really exciting to me.”
	Recruitment by someone other than care provider allows more open communication	The interviewee expresses the idea that having someone other than a care provider could allow more open conversation about what it means to participate	“I think back, I think with her coming in as a not involved third-party doing research I felt that I could talk openly and Mom could talk openly, even the nurse asked if she could stay through the visit and we were fine with that. At least there was not a threat of it wouldn't hinder the care we were receiving at all.”
**DESCRIBING THE IDEAL PATIENT/FAMILY PARTICIPANT**	Used when the interviewee explains the characteristics of the “ideal” patient/family participant in engagement		

	Both personal experience and beyond	The ideal participant is able to go beyond just their own experiences (including generalizability, national advocacy experience, etc.)	“So it's very hard. You need somebody who can speak and be informed by their own experience but who can then carry that to reflect on experiences different than their own. And I have seen this with patient advocates on study sections and such.”
	Willing and able to speak up	The ideal participant is strong enough to be willing and able to speak up in an otherwise intimidating group	“I mean, that's why we wanted her on the team, because she had a voice. She had an opinion and she wasn't shy about saying this is what my experience was and if you want this to be relevant to patients like me here's what you have to do. She was a really great advocate.”
	Knowledge and commitment	The ideal participant has the knowledge and especially the commitment to participate fully	“I would say the characteristics of any panel member, whether it be a patient or an expert, is somebody who's interested in the topic and willing to take the time and-- let me think. Because it is somewhat-- it is a time commitment to do this work, so making sure that they have an understanding of that, and that that's something that they want to do.”
**CHALLENGES OF ENGAGEMENT**	Used when the interviewee reports challenges or factors that can get in the way of effective engagement - here, not explicitly “ethics” challenges, even though some of these have ethical import		
	Challenges engaging a diverse ‘community’	The interviewee describes how diversity in the community makes it hard to know who to engage (e.g., because of widespread geography, other factors)	“So as far as being able to engage people, I think in that sense, I think it's probably pretty similar to the general population. We have 229 tribes in Alaska and a huge amount of cultural diversity.”
	Cultural sensitivity	The interviewee describes the need to culturally tailor the engagement due to how cultural differences including cultural norms surrounding death and dying make it hard to discuss the topic (including taboos discussing people who have died, spiritual beliefs, etc.)	“So yeah, we had very good ethnic diversity, and definitely, there's ways you need to handle different populations so that there's-- sometimes you need cultural translators and that sort of thing. And so we were very kind of aware of that and did do that.”
	Research funding structures constrain engagement	The interviewee describes how certain parts of the research may not be changeable, excluding patients from asking the most fundamental questions	“I just don't think they went about it in a very effective way, and part of the issue was a lot of this was prescribed from work of some other institutions that were part of this consortium. I think there were maybe 10 or 12 other

			institutions and they had already made a lot of decisions about how they wanted to apply a standardized process across all of the cancer centers.”
	Managing issues of death and illness, including progression	The interviewee describes how death and illness experiences of patients and families can create challenges to effective engagement	“And so there were people who had to drop off of the study during that three year period of time because their illness got worse or because they're cognitive function got worse or because their demands as a caregiver became too great.”
	Knowledge	The interviewee describes how lack of knowledge (of health issues, research, and so on) can create a barrier to effective engagement	“One of the practical challenges that I've seen verged into an ethical challenge is that patients and families often don't have a lot of training or background in research. And so I think you have to be very practical about what information, what engagement is good for and how it works. For example, engaging patients and families around the statistical analysis or the questions, the methodological questions about study design or things like that is sort of silly and often not a good use of time. So I think there's sort of this practical challenge of A, how you train people up to play a role and then B, what roles they are best able to play.“
	Time	The interviewee describes how the time required to do engagement can be a challenge	“I mean, I've left a meeting maybe once or twice where I thought not that it didn't go so well, more like, ‘Well, there wasn't enough time for us to cover everything that we were trying to cover.’”
	Money	The interviewee describes how lack of adequate money can limit what can be done regarding engagement	“So, we've been trying to get there, but they haven't been funded yet. So I have submitted several different proposals that have looked at.”
	Minimizing burdens	The interviewee describes how various burdens of participating (e.g., time, travel, caregiving demands) can be a challenge, or that it can be hard to minimize burdents for participants	“Well, yes. I mean, of course, that's a part because in order for me to participate, I'm driving an hour away from home in order to participate, in order to be a part of this, so is it a burden? Yes, but is it worth it? Yes.”

	Lack of familiarity with technology	The interviewee describes how lack of access to technology or know how can limit or affect participation	"Well, as it was, first up she was not very technically savvy so everything had to be a phone call. There never could be any emails."
	How to respond when the patient and caregiver voices are conflicting	The interviewee describes how It can be a challenge when the patient's opinion and the caregiver's opinion differ, making it hard to know which input to take	"And then, of course, it's one of the prognostic factors and one of the things that we don't like to do is dialyze people with progressive or severe dementia because we don't think it's valuable for those patients. And that can be a challenge with the family because they may feel like, "Oh, we want to keep this person alive." And they don't understand the patient can't understand and is suffering, getting dialysis."
	Meeting virtually (i.e. not in person)	The interviewee describes the challenges of meeting using virtual methods including difficulty speaking up, getting comfortable with participation, and so on	"But I prefer to be there in person. It's just easier to watch people respond, and then I can actually hear better that way too. "
	Dealing with different communication abilities (e.g., due to neurological illness)	The interviewee describes how it can be a challenge in certain disease states that create communication difficulties (e.g., neurological diseases)	"So we sort of learned that you have some drawbacks in terms of communication, and maybe thinking about ways to overcome that will be important. I don't know what that would be, I'd have to really give that some thought. But honestly, sometimes depending on-- I know there was one person in particular. He was pretty far along, and most of the time he was pretty easy to understand, but sometimes not."
	Being authentic about engagement	The interviewee describes the broad need to be authentic and true in working with advisors	"But if you're saying that it's important to include those people, have you really? So there's sort of part of this almost facade of showing that you're-- I'm being a little bit brutally honest here-- but facade of showing that you're engaging all these different stakeholders, but are you actually engaging them or is it just that they're sort of present or partially engaged?"
	Fairly compensating participants	The interviewee explains the need to compensate patient/family partners (including in equal amounts to the researchers)	"And so, I think, yeah, it goes beyond just having patient-reported outcomes but having them as really members of the team that are valued and provide input, and insight, and are paid just like

			other research team members, and are part of publications just like the research team is.”
	Defining ownership over data	The interviewee explains how ownership over the products of engagement should be given to the patient/family participants	“Also that the data-- and this goes beyond the CAB, but that the data and the research and the outcomes of the research needs to belong to the community, that the onus of responsibility is on the researcher. But that the ownership of the whole enterprise needs to rest and lie with the community.”
	Avoiding paternalism or gatekeeping	The interviewee describes the ethical concern about ‘gatekeeping’ in EOLPC research - that is, when excluding or not offering participation to certain patients due to their illness	“I think, I believe again, that's part of that paternalism that I was talking about, when we want to protect others from what we might anticipate as harm. That can be reframed, again a good social work word. That can be reframed into the opportunity to have the autonomy to choose, can be seen as a gift, so the person can look you in the eye and say, are you crazy? Of course not. But I get the choice. You didn't preselect me out because of my end-of-life status, or my symptom-burden status, or my whatever it is.”
	Facilitating participation of those with serious illness	The interviewee describes the obligation to minimize burdens to the patient and/or to the caregiver including the obligation to make efforts to give people the chance to participate who otherwise might not be able to	“Yeah. I mean, we would try to meet with people on their own terms. So sometimes people couldn't come in physically; we'd pay for transportation; we'd pay them for their time. If they couldn't come in, we would go to them, or if they were a dialysis patient-- we found that not only the panel but also the patients who were in the study much preferred to meet in the dialysis chair even though it's much less private. They prefer that to spending extra time.”
	Navigating decision capacity or its fluctuation	The interviewee explains that decision making capacity can make it hard to sustain engagement	“Yeah, we had very honest-- from a couple of the patients, they were very honest about how they were dealing with their changes in cognitive ability, two of them, specifically. And these were both men who were in the [name of organization] support group, whom I've known for years, and they were very honest about what's happening, cognitively, with them, how they see changes.”

	Justice and engaging diverse participants	The interviewee explains issues of justice/inclusivity, or how the burdens of participation fall disproportionately on some groups	“But again, to your point, some of this probably is the socioeconomic status of these patients, right? So if you have patients and caregivers who are worried about food and shelter, no matter how valued they feel, you might not be able to engage them because they have larger life issues.”
	Challenges managing participants’ different perspectives	The interviewee describes how diverse participants can have different views, beliefs, expectations, and/or abilities, making it hard to get consensus or balanced feedback	“I mean, we have people - and I'll just say stakeholders - who are in the insurance industry who are multimillionaires, who have very little time. Very challenging to get them to take-- they travel all time. Very challenging to get them to come cross -country just for our meeting. Also, not always the most patient because their time is short and focused, and as committed to this project as they are, they don't necessarily want to hear a patient stakeholder talk about their experience for 30 minutes.”
	Finding the right time to recruit partners, including caregivers	The interviewee explains that recruiting during times of serious illness could be inappropriate	“Especially again as someone who is less experienced and didn't have any cognitive care background, it was difficult to sort of talk about these end-of-life issues. And then of course there's patients who are not feeling well. I was trying to recruit patients in the hospital and you're trying to get them at the end-of-life but they're in acute hospitalization. And then also the closer you get to the end-of-life of course there's issues of capacity and inability to really endure an hour-long or so interview.”
	Managing expectations	The interviewee describes the challenge of ensuring patients/families and researchers share the same expectations regarding engagement	“So thinking that through. I mean, I think again, it has to be just as efficiently run as everything else that you're doing. So you need to have clear expectations, orientation to their group, clear meeting schedule, and probably time limit is going to be for a year or however long, and being consistent about that.”
	Respecting privacy and confidentiality	The interviewee describes issues related to the confidentiality of participants’ information and data obtained during the engagement	“One thing that actually came up with the qualitative interviews came up around confidentiality and some people, even if we had

			de-identified data, could recognize their friends through their stories and support groups.“
	Respecting participants' time	The interviewee describes the obligation to respect participants' time (such as by doing high yield activities when they are present)	"Well, I think trying to basically minimize the amount of-- being very respectful, always, of time. And so if you only need a small amount of time, only use a small amount of time. And to always be on time for meetings and to just generally be respectful of everybody's time, I think, is really, really important."
	Providing opportunities for authorship/credit	The interviewee describes the challenge of authorship norms as they relate to authorship by engagement partners	"And we've, I think, tried our best to include members of our advisory council as authors and to give them credit as a group. But I wonder if we're not saying things the same way we would with colleagues. I think some of our colleagues who are on different papers may have put in less work than some of the people on the advisory council."
	Clinicians recruiting patients they care for	The interviewee explains that clinicians need to be careful when recruiting patients they care for to be engagement partners	"Anytime a clinician is recruiting anyone for something other than clinical care, you have to be very careful about that. I don't ever recruit patients into my own studies, but I know there are people around to do and to say that they couldn't run their studies if they didn't do that."
**RELATIONSHIP ETHICS**	Used when the interviewee describes ethics issues that are outside traditional biomedical ethics - and more about relationships between people		
	Obligation to treat board members as equals	The interviewee describes the obligation to treat patients/families as equal partners	"And I feel like researchers that-- that researchers need to be aware that the-- the participants are their patients should be their primary concern. And it should be the utmost responsibility of the investigators to make sure that they are establishing a community of trust, or relationship of trust, and a relationship that is for lack of a better word, agreeable. That the patient or the participant is going to feel comfortable, and they're going to feel like it's beneficial to them, and they're gonna feel like it's helpful."

	Offering medical advice	The interviewee explains that the researcher can or should offer medical advice when asked or when noticed (e.g., cognitive decline)	"So that's just kind of outside of that whole patient advisory role because these are human beings, you know? And they bring all of their-- like you're in a-- we were in a relationship now, just like any working relationship, the way that a colleague would call you and say, you know. I assume you provide palliative medicine in the hospital or clinic or something, but if you have a colleague, if people know you do palliative care, they're going to call you and say, "My mother just got a diagnosis with blah, blah, blah, what hospice would you recommend?" or "I need help. I need," you know."
	Managing a "bad" partner	The interviewee describes how to deal with engagement when it is going poorly (including terminating relationships with advisors)	"And so, this is where it gets really complicated is people on my staff here were like, "This is completely inappropriate. He's taking advantage of us." There were some other ethical things he did that were really-- he slept through the meeting, didn't participate. So how do you fire a patient stakeholder?"
	Long duration, long journey	The interviewee describes the long-term relationships that form with engagement partners due to the unique setting of EOLPC research (including when relationships extend beyond the conclusion of the research)	"She was a partner and she'd been on this journey with us for a year and change. And I knew she liked visits and she didn't have a lot of family. Her friends were her family. She talked about that a lot. And so we got to know her friends and her friends said they'd loved us to visit. And so remember I brought my sons and my husband to spend an hour drive away, and my son had colored a whole bunch of things that we could decorate her hospice room."
	Friends	The interviewee explains that the relationship between the researcher and the engagement partner can or should be one of friends, or specifically of equal friends	"She is one of the most dearest friends in my life. She is remarkable. She is wonderful. She's compassionate, she's sensitive, she's smart. And she used to always tell me-- what I loved most about her, she said, 'I'll do the research part. I'll do the writing part. You just be you.'"
	Respect	The interviewee explains the need for respect in the researcher/engagement partner relationship	"And I don't know what they could have done to make us feel more valued or whatever. I mean,

			personally, I felt that all of the researchers that I had connections who were very respectful, very much wanting me and us to believe that this was worth our time and this kind of thing.”
	Providing support	The interviewee explains that the research team should provide support to the engagement partners including emotional support (such as when there is loss of a member)	“I got hugs from everyone, and at the end of the meeting, they presented me with a plaque for my mother's contributions to the team. Which I know my mom is so proud of. It has a place of honor on the walls here of her house.”
**DESCRIBING A WAY TO MANAGE A CHALLENGE**	Used when interviewee describes a way to manage a particular challenge or how to do engagement better		
	Have family caregivers involved to support cognitive limitations	The interviewee describes how including a family caregiver can be a way to promote participation of those with cognitive impairment	“Well, yeah. I mean that could be challenging in patients on dialysis in a number of different ways, but having family members who are the caregivers on the panel helped to get the perspective of people who couldn't really talk to us too much or give us too much input.”
	Have short or alternative means of feedback	The interviewee describes the need for researchers to offer alternative methods of feedback that are less demanding of partners	“And I think also, just to re-emphasize that setting out an agenda in advance and giving people a chance to think about things and even write about things offline was pretty important, because a lot of times if people have various cognitive or communication issues that they may not be able to get their thoughts together in an articulate way during the time that we have provided.”
	Include more people	The interviewee describes the need to include more people, which is particularly needed in EOLPC. (e.g., including by having a PCRC repository of engagement participants, a fellowship training program, etc.)	“I do. Yes. I do, because the experiences that people have are very different, and you may hear some things merge with little variation on what the experiences and perspectives are. But I think it would improve the quality of the findings and conclusions if there are more people who participate.”
	Engage patients before they are too sick	The interviewee explains that bringing the patient to the group as an advisor before they are sick can get their voice heard earlier	“I absolutely do. That's why we try to get them before they're there. We just had a memorial service yesterday for one of my cousins who had been sick.”

	Recruit sensitively and unobtrusively	The interviewee describes the need to be sensitive when recruiting patients who are sick or at the end of life (including the idea of having a 3rd party recruit)	"I really think it's certainly okay to ask, and as in anything, it's how it's done, not if it's done I think, and because of their sensitive time that they're in, I'd be asking of be seeking permission and to be as unobtrusive as possible is very important I think."
	Have cultural sensitivity training	The interviewee describes having training on cultural sensitivity for research team members	"I would have everyone that works at the [name of hospital] go through a cultural and linguistic training. Something where they understand the population of patients at [name of hospital]. They understand the demographics. And they understand each culture. They know the differences between each culture. I feel like knowing that they'll be able to connect with their patients a lot more and they'll be able to help them out a lot more."
	Have multiple research sites	The interviewee explains that having multiple sites can get you more diversity and/or more engagement participation	"Well, I think especially for the Native American people, I think they're more familiar with them in New Mexico, and that's very-- they can use cultural translators and-- I wasn't there when they did the panels there, but I think that was one of the issues, and I think they were successful. I think they had good input and honesty from the different ethnic groups that we dealt with, same with the African Americans here, Hispanics."
	Have support services available (e.g., parking)	The interviewee explains the benefits of having support services (including both support for the research team and support for engagement partners)	"Luckily, I'm very well supported in terms of the support services here. So I have an assistant, but she has time to really handcraft a schedule and travel plans and be completely available. She even went to the airport to pick people up."
	Have a peer partner to support the participant	The interviewee describes pairing a patient/family member with someone else to assist them and empower their voice	"So I always make sure that their handler or their partner-- I don't know what you call them-- the study team, the research team partner is there, is one of their first ones that person is seated next to them. And it's empowering them."

	Have strong patient personalities	The interviewee explains how having patient advisors with strong personalities can facilitate participation	“We chose very strong personalities on the patient's side who could stand up to that, and who we knew wouldn't' be intimidated by the payers or the large organizational stakeholders.”
	Understand what voices you need	The interviewee describes the need to understand what voices should be represented on the board (including the idea that not all voices can or should be represented)	“To make sure that everybody's at the table who need to be there. Because it doesn't matter what I think or you think about the situation that's going to impact somebody next door, if the person next door who's having the situation is not at the table.”
	Vet the list of board members	The interviewee encourages the researchers to review and choose thoughtfully the engagement participants in various ways	“And I think, if I were going to-- if I were going to improve on that process at all, one other thing I might do is to then take the list of recommended names, and then ask if there's another person in the health system or in the community in a position of authority who can just verify or confirm or affirm that this is, in fact, the right group of people or the group of people to be in that position.”
	Having a joint patient and caregiver together on the team is useful dialogue	The interviewee describes the value of having both a patient and their caregiver as engagement partners together	“In other words, bringing patients with their caregivers would probably be some form of mitigation against overly burdening a participant.”
	Have a patient Co-I	The interviewee describes how having a patient Co-I creates accountability for the patient to participate	“They were able to have a Co-PI funded PCORI study that I was a part of. But it was literally driven from the patient who had a lived experience and could see where we needed to do differently. And again she was instrumental in pulling together the actual research network.”
	Give time on the agenda	The interviewee expresses the idea that there should be time set aside at meetings to listen to patient/family advisors	“Sometimes we jam way too much into that meeting, and one of the things I've realized is they need time to talk about a project, and they process it through their life experience, and so conversations tend to be very meaningful conversations, and we just need to give available space for it.”
	Set expectations upfront	The interviewee explains the importance of making the expectations of advisors clear from	“So, I mean, we're recruiting for a study now that is caregivers and caregiver-driven. And there's

		the start (including what to expect, how to dress, time commitment, etc.)	some folks who are really enthused, and it's okay for them. We certainly get a fair amount of no's but it's too busy. But does that mean we have to stop asking? I'm not sure. Right. I think we just have to be very clear about what we're expecting of them or what the time commitment is.”
	Share feedback back to participants	The interviewee explains the need to share information about the research back to participants (including specifically about their contributions and impact on the research)	“When we do meet with them, to give them feedback about the ways that their contributions have shaped our study. And so, whenever we work with stakeholders and do an interview with them, we collate the information, we put it in a log, and then we write up the team's response, and then we share that log back with them so they can see how their comments and perspectives were received by the team and how it's changing our work.”
	Use virtual methods to overcome challenges (such as travel)	The interviewee explains how you can use virtual methods to overcome issues such as geographic spread	“They always had a session in person but like four or five months of the year we're in [geographic location] and the meetings are in [geographic location]. So we would do it over the computer. And so that was nice because we could see the group and participate.”
	Seek input in other ways	The interviewee explains how you can get input in ways outside of traditional engagement such as immersion in communities, peer to peer conversations, etc.	“I don't think you can engage in one way. I think one of the big take home lessons that I've learned from this is that having an advisory board is great. You get a certain kind of person but there's lots of kinds of people who aren't going to show up to a meeting like that. And so you have to have-- if you want broad perspectives, you have to have multiple different ways to get those perspectives.”
	More money	The interviewee explains how more funding support could be helpful, particularly for NIH grants that may/may not have engagement funds	“I could improve the process by giving them some more money so that they could upgrade the hardware. And I could improve, although it's probably not possible, the panel by figuring out a way to speed up the various disciplines within the study that require lots of time needed to settle on standardized software and hardware.“

	Conduct evaluation	The interviewee describes using evaluation of the group to inform its function	"So, for example, on one of our evaluations, we got a lot of negative feedback on one particular part just because it was a very technical discussion. And so finding that balance between involving everyone in every step but also meeting individual strengths and interests is something, I think, takes time, over time, and can be achieved."
	Allow additional time for communication	The interviewee explains that investigators should be patient and allow time for engagement partners to provide feedback	"Some people who have Parkinson's, they can be pretty slow to process and also slow to communicate. They may have a low voice. And so I think the cool thing about the council is that everybody on the council understood Parkinson's disease and understood what everyone else was going through and so they were able to shut up and be quiet to give somebody a chance to express themselves who in other groups probably wouldn't have a chance to express themselves because things either moved too fast or people would try to fill in the words for them."
	Have meetings in the community and/or hosted by trusted community organization - at least initially	By placing meetings in the community, at a trusted site, participation can be increased	"I find that when the meeting is set in the communities where people live, you'll probably get a better attendance, and especially if it's linked up either with community organization or church in that community and a place where the people who you want to do the research on are comfortable in their own environment, they feel safe, and they trust the people who are coordinating it."
**DESCRIBING ATTITUDES TOWARD THE ENGAGEMENT** *(added as per patient interviews)*	Used when interviewee expresses their overall attitude/approach to engagement		
	Felt valued	The interviewee describes feeling valued by the research team	"And I have to say, I certainly felt valued. I felt like my interest really was that-- that's what struck a bell with me. The thing about this, this was something that hopefully would benefit patients in the research."
	Worry or uncertainty that contributions didn't make a difference to the research	The interviewee describes feeling as if their participation did not have an impact on the research	"But my true feeling about that is that it likely didn't-- likely it's not going to have a big impact.

			But I don't know that that's the case or not. So that was a little bit of a feeling I was left with it."
	Felt dismissed or not heard	The interviewee describes feeling as if researchers did not listen to them or were dismissive of their contributions	"There weren't enough resources for comments and concerns for improvement. I wish there was a way for caregivers and patients to put more out there and to be - feel like they're being listened to more. Because there's an incredibly isolating feeling."
**DESCRIBING A BENEFIT TO THE ENGAGEMENT PARTNER**	Used when the interviewee describes how the engagement patient/family partner benefits from the activity		
	Giving back	The interviewee describes how the participant felt that they were able to repay or do something in return by being an engagement partner	"As I say, I started to get into the volunteer aspect of it because I wanted to pay back what the hospital did for me. And even though it wasn't successful, while they were able to care for my wife and I just enjoy the challenge and the ability to keep my mind active and to discuss and participate in ventures such as this."
	Giving purpose	The interviewee describes how being an engagement partner can provide a sense of purpose for patients/family especially at the end of life	"It gives my husband a sense of purpose, in terms of dealing with this ugly disease, and that has positive feedback from me, too."
	Learning about research, one's own beliefs, etc.	The interviewee describes how being an engagement partner resulted in the participant learning	"I benefit by learning more about where I thought things were lacking, possibly, and now seeing the bigger picture of it. And then, I benefit by seeing some of the changes put into place."
**DESCRIBING BENEFITS BEYOND THE DIRECT PARTICIPANTS**	Used when the interviewee describes additional benefits beyond the direct participants of engagement		
	Community	The idea that the broader community can benefit indirectly such as via network connections	"So I represented my race because that's what they needed. They needed an African American who was a member of an African American church. They were looking for African Americans to be a part of their research. I was the only African American on that team, and it was a great experience, for me, to work on what I call a think

			tank and to see how someone taking a very small idea and in doing research and how it develops. So I was fascinated, amazed, and honored to have been able to participate in that and because of it I was able to share it with my siblings, my children, my husband, my church community, and my race. So it was bigger than me, it was not even about me."
	Patients or family members, but not the direct participant	The idea that the patients or family members beyond the individual engagement participant may benefit	"But in the meantime, I had a sister at the time, who was a dialysis patient and had a number of different chronic diseases, who became very excited about what was going on. And she decided that she wanted to learn more, and she wanted to participate. And then they have an educational program."
	Spreads the research message	The idea that engagement helps spread the research message	"Well, I would try to describe like you did the benefits of the study. How it's going to be helpful to find out what the answers to your questions. And it's going to help a lot of patients and caregivers down the road. And that's what we all felt about what we were doing before. You're making a big impact on the whole field of Parkinson's treatment, movement disorders. So it's her chance to have a voice and make an impact."
**DESCRIBING A BENEFIT TO THE INVESTIGATOR**	Used when the interviewee describes how engagement benefited the investigator		
**DESCRIBING a NEGATIVE EFFECT OF ENGAGEMENT ON THE PARTNERS**	Used when interviewee describes something bad that comes about because of engagement		
	Contributes to bereavement	The interviewee explains how participating in engagement can potentially contribute to bereavement of the engagement partner	"I think the risk is not just that you might get upset, but it could potentially open up all of these issues for people. And do you have really good potentially psychiatric support available for people, or what do you do if they disclose really, really concerning experiences?"

	Discomfort from learning about their future with their illness	The interviewee describes discomfort felt by a patient partner who must confront their future with their illness due to being on a board with other patients who are further along in their disease progression	"And specifically, one of our patients, he was glad to have it. But he just thought it was, in some ways, kind of a difficult pill to swallow, to learn about red flags for hospice for people with Parkinson's disease and what his future might look like."
**PATIENTS' / FAMILIES' VIEWS OF WHO THEY REPRESENT**	Used when the interviewee describes whom they think they "represent"		
	Race	The interviewee describes feeling as though they represent their entire race while being an engagement partner	"So I represented my race because that's what they needed. They needed an African American who was a member of an African American church."
**DESCRIBING THE ROLE OF THE CAREGIVER PARTNER**	Used when the interviewee discusses whether the caregiver is a surrogate for the patient or is to express views as a caregiver		
	Both	The interviewee describes feeling as though the caregiver represents both themself and the patient	"I would say a combination of both. I'm not saying us versus them in patients or anything. It's because we're with the patient 24/7. I guess it's a challenge that both the patient and the caregiver sees because I feel like, in a lot of ways, the caregiver gets the double the challenges."
	Caregiver only	The interviewee describes feeling as though the caregiver represents only the caregiver voice	"Mostly mine as a caregiver. There would be times we talked about needs at certain stages or something, and I could remember things that we went through with him. But it was mostly me as a caregiver. Ever since I got on that council I kind of considered myself a caregiver advocate."
**CRITICIZING THE STATUS QUO**	Used when the interviewee criticizes how engagement gets done, or the view that it's not important		
