# Supplementary material for: Meeting ethical challenges with authenticity when engaging patients and families in end-of-life and palliative care research: a qualitative study
Source: BMC Palliat Care. 2022 May 16;21:74. doi: 10.1186/s12904-022-00964-x (PMC9108140; doi:10.1186/s12904-022-00964-x)
Supplement: Supplementary file 2 — Additional file 2. [file 12904_2022_964_MOESM2_ESM.pdf]

Appendix 2. Challenges and potential solutions identified in interviews (Numbers correspond to instances of coding; I = investigator interviews; P&FC = patient and family caregiver interviews)

CHALLENGES	CODES PRESENT			POTENTIAL SOLUTIONS	CODES PRESENT		
	I	P&FC	Total		I	P&FC	Total
**Justice and engaging diverse participants**	32	25	57	**Understand what voices you need**	1	3	4
				**Include more people**	2	4	6
**Facilitating participation of those with serious illness**	29	12	41	**Have meetings in the community and/or hosted by trusted community organization - at least initially.**	1	2	3
				**Use virtual methods to overcome certain challenges (such as travel)**	2	10	12
**Managing issues of death and illness, including progression**	23	8	31	**Engage patients before they are too sick**	1	1	2
**Avoiding paternalism or gatekeeping**	12	5	17	**Recruit sensitively and unobtrusively**	5	9	14
**Meeting virtually (i.e. not in person)**	3	14	17	**Have strong patient personalites**	1	0	1
**Minimizing burdens**	5	10	15	**Have support services available (e.g., parking)**	2	2	4

**Respecting privacy and confidentiality**	5	9	14	**Set expectations upfront**	19	11	30
**Managing Expectations**	10	2	12	**Set expectations upfront**	19	11	30
**Navigating decision capacity or its fluctuation**	7	2	9	**Have family caregivers involved to support cognitive limitations**	3	1	4
**Fairly compensating participants**	7	2	9	**More money**	1	1	2
**Knowledge**	4	4	8	**Vet the list of board members**	2	3	5
**Respecting participants' time**	2	6	8	**Give time on the agenda**	1	1	2
**Clinicians recruiting patients they care for**	7	0	7	**Recruit sensitively and unobtrusively**	5	9	14
**Dealing with different communication abilities (e. g., due to neurological illness)**	3	4	7	**Allow additional time for communication**	1	0	1
				**Have short or alternative means of feedback**	7	11	18
				**Have a peer partner to support the participant**	3	1	4
**Being authentic about engagement**	2	4	6	**Share feedback back to participants**	5	3	8

**Finding the right time to recruit partners, including caregivers**	2	4	6	**Recruit sensitively and unobtrusively**	5	9	14
**Time**	1	3	4	**Set expectations upfront**	19	11	30
				**Seek input in other ways**	3	1	4
**Providing opportunities for authorship/credit**	3	0	3	**Have a patient Co-I**	1	0	1
				**Set expectations upfront**	19	11	30
**Challenges managing participants' different perspectives**	2	1	3	**Have short or alternative means of feedback**	7	11	18
				**Have strong patient personalites**	1	0	1
				**Set expectations upfront**	19	11	30
**Cultural sensitivity**	3	0	3	**Have cultural sensitivity training**	0	1	1
**How to respond when the patient and caregiver voices are conflicting**	3	0	3	**Having a joint patient and caregiver together on the team is useful dialogue**	2	1	3
**Lack of familiarity with technology**	2	1	3	**Vet the list of board members**	2	3	5

**Money**	1	1	2	**More money**	1	1	2
**Defining ownership over data**	1	0	1	**Set expectations upfront**	19	11	30
				**Share feedback back to participants**	5	3	8
**Challenges engaging a diverse 'community'**	1	0	1	**Have multiple research sites**	3	2	5
**Research funding structures constrain engagement**	0	1	1	**More money**	1	1	2
**Totals**	174	119	293	**Totals**	68	68	136
